# Supplementary material for: Encountering Parents Who Are Hesitant or Reluctant to Vaccinate Their Children: A Meta-Ethnography
Source: Int J Environ Res Public Health. 2021 Jul 16;18(14):7584. doi: 10.3390/ijerph18147584 (PMC8306550; doi:10.3390/ijerph18147584)
Supplement: Supplementary file 1 [file ijerph-18-07584-s001.zip › ijerph-1268507-supplementary.pdf]

Supplementary Materials

Table S1. eMERGe Guideline

No.	Criteria Headings	Reporting Criteria	Meta-ethnography – criteria met (Page No)
**Phase 1—Selecting meta-ethnography and getting started**			
Introduction			
1	Rationale and context for the meta-ethnography	Describe the gap in research or knowledge to be filled by the meta-ethnography, and the wider context of the meta-ethnography	Page 1-2
2	Aim(s) of the meta-ethnography	Describe the meta-ethnography aim(s)	Page 2
3	Focus of the meta-ethnography	Describe the meta-ethnography review question(s) (or objectives)	Page 2
4	Rationale for using meta-ethnography	Explain why meta-ethnography was considered the most appropriate qualitative synthesis methodology	Pages 2; 17-18
**Phase 2—Deciding what is relevant**			
Methods			
5	Search strategy	Describe the rationale for the literature search strategy	Page 2
6	Search processes	Describe how the literature searching was carried out and by whom	Page 2 -3
7	Selecting primary studies	Describe the process of study screening and selection, and who was involved	Page 2 -3
Findings			
8	Outcome of study selection	Describe the results of study searches and screening	Page 13
**Phase 3—Reading included studies**			
Methods			
9	Reading and data extraction approach	Describe the reading and data extraction method and processes	Page 4-12
Findings			
10	Presenting characteristics of included studies	Describe characteristics of the included studies	Page 13, Table 2
**Phase 4—Determining how studies are related**			
Methods			

11	Process for determining how studies are related	Describe the methods and processes for determining how the included studies are related:<br>- Which aspects of studies were compared<br>AND<br>- How the studies were compared	Page 4-12
Findings			
12	Outcome of relating studies	Describe how studies relate to each other	Pages 13-16
**Phase 5—Translating studies into one another**			
Methods			
13	Process of translating studies	Describe the methods of translation**:**<br>- Describe steps taken to preserve the context and meaning of the relationships between concepts within and across studies- Describe how the reciprocal and refutational translations were conducted- Describe how potential alternative interpretations or explanations were considered in the translations	Pages 4 - 12
Findings			
14	Outcome of translation	Describe the interpretive findings of the translation.	Pages 13-16
**Phase 6—Synthesizing translations**			
Methods			
15	Synthesis process	Describe the methods used to develop overarching concepts ("synthesised translations") Describe how potential alternative interpretations or explanations were considered in the synthesis	Page 4-12
Findings			
16	Outcome of synthesis process	Describe the new theory, conceptual framework, model, configuration, or interpretation of data developed from the synthesis	Pages 13-16
**Phase 7—Expressing the synthesis**			
Discussion			
17	Summary of findings	Summarize the main interpretive findings of the translation and synthesis and compare them to existing literature	Pages 16 - 18
18	Strengths, limitations, and reflexivity	Reflect on and describe the strengths and limitations of the synthesis:<br>- Methodological aspects—for example, describe how the synthesis findings were influenced by the nature of the included studies and how the meta-ethnography was conducted.- Reflexivity—for example, the impact of the research team on the synthesis findings	Pages 17 - 18

19	Recommendations and conclusions	Describe the implications of the synthesis	Pages 16 - 18

Table S2. Search strategy

**Pubmed**		PubMed
#1	(("Parents"[Mesh] OR "Nurses"[Mesh] OR "Health Personnel"[Mesh]) AND (perspective*))	22,229
#2	view* [tw]	436,820
#3	opinion* [tw]	93,037
#4	experienc* [tw]	1,020,920
#5	"Attitude"[Mesh]	538,634
#6	“Know” [tw]	82,980
#7	OR #1 - #6	1,980,211
	**(("Parents"[Mesh] OR "Nurses"[Mesh] OR "Health Personnel"[Mesh]) AND (perspective*)) [tw] OR view* [tw] OR opinion* [tw] OR experienc* [tw] OR "Attitude"[Mesh] OR “Know” [tw]**	
#8	"Health Personnel"[Mesh]	458,885
#9	"Physicians"[Mesh]	120,596
#10	"Primary Health Care"[Mesh]	147,051
#11	"Nurses"[Mesh]	79,717
#12	"Nursing Care"[Mesh]	113,436
#13	"Nursing"[Mesh]	211,270
#14	"Nurse's Role"[Mesh]	37,960
#15	"Physician's Role"[Mesh]	26,695
#16	"Nurse-Patient Relations"[Mesh]	28,859
#17	"Professional-Patient Relations"[Mesh]	125,497
#18	"Parents"[Mesh]	104,160
#19	OR #8 - #18	952,863
	**"Health Personnel"[Mesh] OR "Physicians"[Mesh] OR "Primary Health Care"[Mesh] OR "Nurses"[Mesh] OR "Nursing Care"[Mesh] OR "Nursing"[Mesh] OR "Nurse's Role"[Mesh] OR "Physician's Role"[Mesh] OR "Nurse-Patient Relations"[Mesh] OR "Professional-Patient Relations"[Mesh] OR "Parents"[Mesh]**	
#20	(((vaccin* OR immunis* OR immuniz*) AND (anti-vaccin* OR Rejection OR refus* OR mandatory OR criticis* OR hesitanc* OR doubt* OR refrain OR opposition))	9,515
#21	"Anti-Vaccination Movement"[Mesh]	51
#22	"Vaccination Refusal"[Mesh]	237
#23	"non-vaccination" [tw]	320
#24	OR #23 - #26	9,839
	**(((vaccin* OR immunis* OR immuniz*) AND (anti-vaccin* OR Rejection OR refus* OR mandatory OR criticis* OR hesitanc* OR doubt* OR refrain OR opposition)) OR "Anti-Vaccination Movement"[Mesh] OR "Vaccination Refusal"[Mesh] OR "non-vaccination" [tw])**	
#25	"Qualitative Research" [Mesh]	50,914
#26	"qualitative" [tw]	230,087
#27	“phenomenolog*” [tw]	25,174

#28	“grounded theory” [tw]	11,177
#29	“ethnograph*” [tw]	10,460
#30	“interview*” [tw]	364,446
#31	“focus group*” [tw]	49,031
#32	"Narration"[Mesh]	7,972
#33	"narrative*" [tw]	38,612
#34	OR #25 - #33	579,024
**"Qualitative Research" [Mesh] OR "qualitative" [tw] OR “phenomenolog*” [tw] OR “grounded theory” [tw] OR “ethnograph*” [tw] OR “interview*” [tw] OR “focus group*” [tw] OR "Narration"[Mesh] OR "narrative*" [tw]**		
#7 AND #19 AND #24 AND #34<br>Limits: Language: English, Portuguese and Spanish.		
**((((("Parents"[Mesh] OR "Nurses"[Mesh] OR "Health Personnel"[Mesh]) AND (perspective*)) [tw] OR view* [tw] OR opinion* [tw] OR experienc* [tw] OR "Attitude"[Mesh] OR “Know” [tw])) AND ("Health Personnel"[Mesh] OR "Physicians"[Mesh] OR "Primary Health Care"[Mesh] OR "Nurses"[Mesh] OR "Nursing Care"[Mesh] OR "Nursing"[Mesh] OR "Nurse's Role"[Mesh] OR "Physician's Role"[Mesh] OR "Nurse-Patient Relations"[Mesh] OR "Professional-Patient Relations"[Mesh] OR "Parents"[Mesh])) AND (AND (((vaccin* OR immunis* OR immuniz*) AND (anti-vaccin* OR Rejection OR refus* OR mandatory OR criticis* OR hesitanc* OR doubt* OR refrain OR opposition)) OR "Anti-Vaccination Movement"[Mesh] OR "Vaccination Refusal"[Mesh] OR "non-vaccination" [tw]))) AND ("Qualitative Research" [Mesh] OR "qualitative" [tw] OR “phenomenolog*” [tw] OR “grounded theory” [tw] OR “ethnograph*” [tw] OR “interview*” [tw] OR “focus group*” [tw] OR "Narration"[Mesh] OR "narrative*" [tw])**		
**TOTAL: 211**		

**SCOPUS**	Scopus	
#1	TITLE-ABS-KEY (“perspective*”)	1,000,591
#2	TITLE-ABS-KEY (“view*”)	1,550,463
#3	TITLE-ABS-KEY (“opinion*”)	241,674
#4	TITLE-ABS-KEY (“experienc*”)	2,152,844
#5	TITLE-ABS-KEY (“attitude*”)	850,339
#6	TITLE-ABS-KEY (“know”)	271,848
#7	OR #1 - #6	5,312,936
**(TITLE-ABS-KEY (perspective* OR view* OR opinion* OR experienc* OR attitude* OR “Know”))**		
#8	TITLE- ABS-KEY (“health personnel”)	155,498
#9	TITLE-ABS-KEY (“health care provider*”)	39,333
#10	TITLE-ABS-KEY (“primary health care”)	99,180
#11	TITLE-ABS-KEY (“physician*”)	637,094
#12	TITLE-ABS-KEY (“nurs*”)	732,387
#13	TITLE-ABS-KEY (”nursing care”)	48,413
#14	TITLE-ABS-KEY (”nurs* role”)	43,330
#15	TITLE-ABS-KEY (”physician* role”)	23,728
#16	TITLE-ABS-KEY (”professional-patient relations*”)	34,543
#17	TITLE-ABS-KEY (”parents”)	519,894
#18	OR #8 - #17	1,970,031
**(TITLE-ABS-KEY ("Health Personnel" OR “health care provider*” OR "Primary Health Care" OR "Physician*" OR Nurs* OR "Nursing Care" OR "Nurs* Role" OR "Physician* Role" OR "Professional-Patient Relations*” OR "Parents”))**		
#19	TITLE-ABS-KEY (vaccin* OR immunis* OR immuniz*) AND (anti-vaccin* OR Rejection OR refus* OR mandatory OR criticis* OR hesitanc* OR doubt* OR refrain OR opposition)	29,249

#20	TITLE-ABS-KEY (“anti-vaccination movement*”)	192
#21	TITLE-ABS-KEY (“non-vaccination*”)	337
#22	OR #19 - #21	29,490
**(TITLE-ABS-KEY (((vaccin* OR immunis* OR immuniz*) AND (anti-vaccin* OR Rejection OR refus* OR mandatory OR criticis* OR hesitanc* OR doubt* OR refrain OR opposition)) OR "Anti-Vaccination Movement*" OR "non-vaccination*"))**		
#23	TITLE-ABS-KEY (“qualitative research”)	108,963
#24	TITLE-ABS-KEY (“qualitative”)	626,075
#25	TITLE-ABS-KEY (“phenomenolog*”)	126,659
#26	TITLE-ABS-KEY (“grounded theory”)	22,689
#27	TITLE-ABS-KEY (“ethnograph*”)	70,737
#28	TITLE-ABS-KEY (“interview*”)	694,922
#29	TITLE-ABS-KEY (“focus group”*)	83,652
#30	TITLE-ABS-KEY (“narration”)	14,256
#31	TITLE-ABS-KEY (“narrative”*)	167,635
#32	OR #23 - #31	1,502,863
**(TITLE-ABS-KEY ("Qualitative Research" OR "qualitative" OR "phenomenolog*" OR "grounded theory" OR "ethnograph*" OR "in-terview*" OR "focus group*" OR "Narration" OR "narrative*"))**		
TITLE-ABS-KEY #7 AND TITLE-ABS-KEY #18 AND TITLE-ABS-KEY #22 AND TITLE-ABS-KEY #32<br>Limits: Language: English, Portuguese and Spanish.		
**(TITLE-ABS-KEY (perspective* OR view* OR opinion* OR experienc* OR attitude* OR "Know")) AND (TI-TLE-ABS-KEY ("Health Personnel" OR "health care provider*" OR "Primary Health Care" OR "Physician*" OR nurs* OR "Nursing Care" OR "Nurs* Role" OR "Physician* Role" OR "Professional-Patient Relations*" OR "Parents")) AND (TITLE-ABS-KEY (((vaccin* OR immunis* OR immuniz*) AND (anti-vaccin* OR rejection OR refus* OR mandatory OR criticis* OR hesitanc* OR doubt* OR refrain OR opposition)) OR "Anti-Vaccination Move-ment*" OR "non-vaccination*")) AND (TITLE-ABS-KEY ("Qualitative Research" OR "qualitative" OR "phenome-nolog*" OR "grounded theory" OR "ethnograph*" OR "interview*" OR "focus group*" OR "Narration" OR "nar-rative*"))**		
**TOTAL: 335**		

**CINAHL**		
#1	“perspective*”	119,996
#2	“view*”	113,755
#3	“opinion*”	36,918
#4	“experienc*”	399,159
#5	(MH "Attitude of Health Personnel")	39,379
#6	(MH "Parental Attitudes")	11,114
#7	“Know”	37,524
#8	OR TX #1 - #7	935,084
**TX perspective* OR view* OR opinion* OR experienc* OR (MH "Attitude of Health Personnel") OR (MH "Parental Attitudes") OR “Know”**		
#9	(MH "Nurses")	55,762
#10	"Nursing"	608,114
#11	(MH "Nursing Role")	49,300
#12	(MH "Nurse-Patient Relations")	23,569
#13	(MH "Professional-Patient Relations+")	83,515
#14	(MH "Health Personnel+")	500,601
#15	(MH "Physicians")	54,263
#16	(MH "Parents")	38,262
#17	OR TX #9 - #16	1,037,263
**TX (MH "Nurses") OR "Nursing" OR (MH "Nursing Role") OR (MH "Nurse-Patient Relations") OR (MH "Professional-Patient Relations+") OR (MH "Health Personnel+") OR (MH "Physicians") OR (MH "Parents")**		
#18	((vaccin* OR immunis* OR immuniz*) AND (anti-vaccin* OR refus* OR Rejection OR mandatory OR criticis* OR hesitanc* OR doubt* OR refrain OR opposition))	2,095
#19	(MH "Anti-Vaccination Movement")	73
#20	"non vaccination"	349
#21	OR TX #18 - #20	2,156
**TX ((vaccin* OR immunis* OR immuniz*) AND (anti-vaccin* OR refus* OR Rejection OR mandatory OR criticis* OR hesitanc* OR doubt* OR refrain OR opposition)) OR (MH "Anti-Vaccination Movement") OR "non vaccination"**		
#22	(MH "Qualitative Studies+")	131,545
#23	"qualitative"	153,274

#24	(MH "Interviews+")	202,760
#24	(MM "Focus Groups")	698
#25	(MH "Narratives")	16,099
#26	OR TX #22 - #25	297,525
**TX**	**(MH "Qualitative Studies+") OR "qualitative" OR (MH "Interviews+") OR (MM "Focus Groups") OR (MH "Narratives")**	
	TX #8 AND TX #17 AND TX #21 AND TX #26<br>Limits: Language: English and Portuguese.	
**TX**	**perspective* OR view* OR opinion* OR experienc* OR (MH "Attitude of Health Personnel") OR (MH "Parental Attitudes") OR “Know” AND TX (MH "Nurses") OR "Nursing" OR (MH "Nursing Role") OR (MH "Nurse-Patient Relations") OR (MH "Professional-Patient Relations+") OR (MH "Health Personnel+") OR (MH "Physicians") OR (MH "Parents") AND TX ((vaccin* OR immunis* OR immuniz*) AND (anti-vaccin* OR refus* OR Rejection OR mandatory OR criticis* OR hesitanc* OR doubt* OR refrain OR opposition)) OR (MH "Anti-Vaccination Movement") OR "non vaccination" AND TX (MH "Qualitative Studies+") OR "qualitative" OR (MH "Interviews+") OR (MM "Focus Groups") OR (MH "Narratives")**	
	**TOTAL: 92**	

**WEB OF SCIENCE**		
#1	“perpective*”	275
#2	“view*”	5,034,649
#3	“opinion*”	162,215
#4	“experienc*”	1,713,296
#5	“attitude”	117,985
#6	“know”	170,983
#7	OR #1 - #6	3,059,918
**TX**	**(perspective* OR view* OR opinion* OR experienc* OR attitude OR “Know”)**	
#8	"Health Personnel"	160,915
#9	“health care provider*”	40,014
#10	"Primary Health Care"	98,979
#11	"Physician*"	603,965
#12	Nurs*	799,841
#13	"Nursing Care"	47,246
#14	"Nurs* Role"	3,088
#15	"Physician* Role"	709
#16	"Professional-Patient Relations*”	38,459
#17	"Parents”	300,649
#18	OR #8 - #17	1,798,944
**TX**	**("Health Personnel" OR “health care provider*” OR "Primary Health Care" OR "Physician*" OR Nurs* OR "Nursing Care" OR "Nurs* Role" OR "Physician* Role" OR "Professional-Patient Relations*” OR "Parents”)**	
#19	((vaccin* OR immunis* OR immuniz*) AND (anti-vaccin* OR Rejection OR refus* OR mandatory OR criticis* OR hesitanc* OR doubt* OR refrain OR opposition))	16,645
#20	"Anti-Vaccination Movement"	113
#21	"non-vaccination"	383
#22	OR #19 - #21	16,990
**TX**	**(((vaccin* OR immunis* OR immuniz*) AND (anti-vaccin* OR Rejection OR refus* OR mandatory OR criticis* OR hesitanc* OR doubt* OR refrain OR opposition)) OR "Anti-Vaccination Movement" OR "non-vaccination")**	
#23	"Qualitative Research"	96,230
#24	"qualitative"	570,551
#24	“phenomenolog*”	187,441
#25	“grounded theory”	20,838
#26	“ethnograph*”	61,151
#27	“interview*”	643,788
#28	“focus group*”	78,142
#29	"Narration”	14,110
#30	"narrative*"	161,139
#31	OR #23 - #30	1,451,788
**TX**	**("Qualitative Research" OR "qualitative" OR “phenomenolog*” OR “grounded theory” OR “ethnograph*” OR “interview*” OR “focus group*” OR "Narration” OR "narrative*")**	
	TEMA: #7 AND TEMA: #18 AND TEMA: #22 AND TEMA: #31<br>Limits: Language: English, Portuguese and Spanish.	
**TX**	**(perspective* OR view* OR opinion* OR experienc* OR attitude OR “Know”) AND TX ("Health Personnel" OR “health care provider*” OR "Primary Health Care" OR "Physician*" OR Nurs* OR "Nursing Care" OR "Nurs* Role" OR "Physician* Role" OR "Professional-Patient Relations*” OR "Parents”) AND TX (((vaccin* OR immunis* OR immuniz*) AND (anti-vaccin* OR**	

**Rejection OR refus* OR mandatory OR criticis* OR hesitanc* OR doubt* OR refrain OR opposition)) OR "Anti-Vaccination Movement" OR "non-vaccination") AND TX ("Qualitative Research" OR "qualitative" OR "phenomenolog*" OR "grounded theory" OR "ethnograph*" OR "interview*" OR "focus group*" OR "Narration" OR "narrative*")**
**TOTAL: 368**

**PSYCINFO**		
#1	(("Parent*" OR "Nurs*" OR "professional") AND (perspective*))	43,137
#2	view*	210,107
#3	opinion*	49,587
#4	experienc*	470,546
#5	attitude	416,475
#6	"Know"	32,111
#7	OR #1 - #6	1,003,164
**(("Parent*" OR "Nurs*" OR "professional") AND (perspective*)) OR view* OR opinion* OR experienc* OR attitude OR "Know"**		
#8	"Health Personnel"	89,114
#9	"health care provider*"	9,042
#10	"Primary Health Care"	23,374
#11	"Physician*"	83,556
#12	Nurs*	141,360
#13	"Nursing Care"	4,935
#14	"Nurs* Role"	699
#15	"Physician* Role"	101,816
#16	"Professional-Patient Relations*"	9,664
#16	"Parents"	128,824
#17	OR #8 - #16	409,235
**"Health Personnel" OR "health care provider*" OR "Primary Health Care" OR "Physician*" OR Nurs* OR "Nursing Care" OR "Nurs* Role" OR "Physician* Role" OR "Professional-Patient Relations*" OR "Parents"**		
#18	((vaccin* OR immunis* OR immuniz*) AND (anti-vaccin* OR Rejection OR refus* OR mandatory OR criticis* OR hesitanc* OR doubt* OR refrain OR opposition))	425
#19	"Anti-Vaccination Movement"	6
#20	"non-vaccination"	24
#21	OR #18 - #20	442
**(((vaccin* OR immunis* OR immuniz*) AND (anti-vaccin* OR Rejection OR refus* OR mandatory OR criticis* OR hesitanc* OR doubt* OR refrain OR opposition)) OR "Anti-Vaccination Movement" OR "non-vaccination")**		
#22	"Qualitative Research"	29,366
#23	"qualitative"	215,791
#24	"phenomenolog*"	23,139
#25	"grounded theory"	9,186
#26	"ethnograph*"	17,912
#27	"interview*"	406,198
#28	"focus group*"	36,180
#29	"Narration"	4,040
#30	"narrative*"	42,720
#31	OR #22 - #30	541,625
**"Qualitative Research" OR "qualitative" OR "phenomenolog*" OR "grounded theory" OR "ethnograph*" OR "interview*" OR "focus group*" OR "Narration" OR "narrative*"**		
#7 AND #17 AND #21 AND #31<br>Limits: Scientific Magazine and Language: English, Portuguese and Spanish.		
**(("Parent*" OR "Nurs*" OR "professional") AND (perspective*)) OR view* OR opinion* OR experienc* OR attitude OR "Know" AND "Health Personnel" OR "health care provider*" OR "Primary Health Care" OR "Physician*" OR Nurs* OR "Nursing Care" OR "Nurs* Role" OR "Physician* Role" OR "Professional-Patient Relations*" OR "Parents" AND (((vaccin* OR immunis* OR immuniz*) AND (anti-vaccin* OR Rejection OR refus* OR mandatory OR criticis* OR hesitanc* OR doubt* OR refrain OR opposition)) OR "Anti-Vaccination Movement" OR "non-vaccination") AND "Qualitative Research" OR "qualitative" OR "phenomenolog*" OR "grounded theory" OR "ethnograph*" OR "interview*" OR "focus group*" OR "Narration" OR "narrative*"**		
**TOTAL: 54**		

Table S3 Traslation tables

**Stone Hardness—Resistance to Vaccination**											
**(Rudolfsson & Karlsson, 2019)**	**(Stretch et al., 2009)**	**(Omura et al., 2014)**	**(Jama et al., 2019)**	**(Khan & Sahibzada, 2016) ⚜**	**(L. Mollema et al., 2012)**	**(Ruijs et al., 2012)**	**(Maria Gottvall et al., 2011)**	**(Shahbari et al., 2020)**	**(Navin et al., 2019)**	**(Berry et al., 2017)**	**(Bašnáková & Hatoková, 2017)**
Explain that there is false information about the autism-vaccine relationship.			False belief of autism Parents tell them that they do not want to administer vaccines that make their children stop talking.								
				Influence of religion Parents listened to religious leaders, who told them that the vaccines were made with the blood of animals prohibited by Islam.	Linked to confidence in homeopathy.				Simulate the behavior of Jesus (who was not vaccinated) and in God's plan. Parents cannot be underestimated from these consultations.	Trust in traditional or complementary medicine. Greater confidence in these "sciences" than in medicine.	

					Strong influence of the pharmaceutical industry and economic interest in changes in immunization programs.					Very harsh tone, compromised health worker safety.	Influence of pharmaceutical companies. Brochures offered to parents to inform them of the facts by large companies.
**Lack of Modelling Tools—Lack of Resources, Support and Training**											
**(Rudolfsson & Karlsson, 2019)**	**(Stretch et al., 2009)**	**(Omura et al., 2014)**	**(Jama et al., 2019)**	**(Khan & Sahibzada, 2016) ⚜**	**(L. Mollema et al., 2012)**	**(Ruijs et al., 2012)**	**(Maria Gottvall et al., 2011)**	**(Shahbari et al., 2020)**	**(Navin et al., 2019)**	**(Berry et al., 2017)**	**(Bašnáková & Hatoková, 2017)**
Lack of training of professionals on vaccination. Lack of training with well-trained parents.					Need for up-to-date training on vaccines, and global epidemics. Know how to anticipate questions from parents.		Lack of knowledge about the HPV vaccine. Nurses request the same guidelines for all.				

	Many changes in some vaccines.		Constant changes in vaccination programs. Alternative calendars
Little coordination between doctors and public health. Loss of commitment to physicians regarding vaccination	Vaccine staff have their own opinion about vaccines and changes, but are not asked. They have to accept everything.	Lack of recognition of health professionals . Desire to be involved in the planning of vaccination programs. Nursing own voice.	
Lack of time and high workload. Process that takes a long time, informing parents, vaccinating, answering questions, examining a minor, etc ...	Little time to talk to parents about vaccines in the consultation. Need to do health checks for the elderly.		

		Unfair economic remuneration.									

Rudimentary Sculpting—Using Personal Strategies

**(Rudolfsson & Karlsson, 2019)**	**(Stretch et al., 2009)**	**(Omura et al., 2014)**	**(Jama et al., 2019)**	**(Khan & Sahibzada, 2016) ⚜**	**(L. Mollema et al., 2012)**	**(Ruijs et al., 2012)**	**(Maria Gottvall et al., 2011)**	**(Shahbari et al., 2020)**	**(Navin et al., 2019)**	**(Berry et al., 2017)**	**(Bašnáková & Hatoková, 2017)**
	Personal conflict between the personal and professional part. Nurses who would not vaccinate their children.				Management of responsibility and feelings for professionals. One cannot be a health worker and be against vaccines.			Differences of opinion in some doctors, who were against.			Handling personal failure after failing to get vaccinated with hesitant parents.
Avoid judging and criticizing the ideas and opinions of parents. Accept their decision	Vaccines are positive, but it is necessary to listen to parents who reject them.				Respect the parents' decision, and have empathy.				Importance of respecting the values of parents, even when they refuse to vaccinate.		Provide enough information to the parents and then leave the final decision

even if they are against it.	The opinions of the parents must be valued.										to the parents.
	I do not wish to break trust with parents, as they will have to deal with those parents in the future.				Establishment of a doctor-patient trust relationship for all health plans, including vaccines.					Related to the maintenance of the therapeutic relationship. Even forgo vaccination if the relationship is at risk.	Lack of trust affects all parental decisions. Depending on the type of parents, one strategy or another will be chosen
						Provide medical information. Explain the risks of diseases, adverse effects, and the positive effect of vaccination.					
